# *Silybum marianum* Extract: A Highly Effective Natural Alternative to Retinoids to Prevent Skin Aging Without Side Effects

**DOI:** 10.1111/jocd.16613

**Published:** 2024-12-18

**Authors:** Cloe Boira, Emilie Chapuis, Laura Lapierre, Jean Tiguemounine, Amandine Scandolera, Romain Reynaud

**Affiliations:** ^1^ Science and Technology Givaudan France SAS Pomacle France; ^2^ Plastic Surgery Polyclinic of Courlancy Reims France

**Keywords:** rejuvenation, retinoids alternative, *Silybum marianum*, skin aging

## Abstract

**Background:**

Vitamin A, or retinol, is one of the most effective antiaging molecules, but it presents issues with photo‐sensitivity and irritation. Alternatives are emerging, but have so far been less effective.

**Objective:**

Here, we present a *Silibum marianum* extract (SME) as a retinol‐like ingredient providing both safety and efficacy. SME was compared to the reference compound, retinol, and to the main alternative, bakuchiol.

**Methods:**

Skin explants from a 58‐year‐old donor were treated with pure retinol (0.1%), bakuchiol (0.2%), or SME (0.8%). After 5 days, collagen and hyaluronic acid levels were analyzed. A placebo‐controlled study involving 57 volunteers was also conducted, with products applied twice daily for 56 days. Results were measured by AEVA‐HE and VISA.

**Results:**

Levels of collagen III were significantly increased by SME, by 23% and 16% compared to bakuchiol and retinol respectively. Compared to bakuchiol, SME treatment increased hyaluronic acid production by 36%. In clinical tests, SME had a significantly stronger anti‐wrinkle effect than bakuchiol—reducing the number of wrinkles on the forehead by 21% and their circumference by 17%—producing effects similar to retinol, and better than bakuchiol. In the self‐assessment, 43% of volunteers reported discomfort while using retinol compared to 0% for the SME formulation. By enhancing levels of collagen III—the youth collagen—and hyaluronic acid in the skin, SME paves the way for the maturation of collagen I fibrils and skin plumping.

**Conclusion:**

With its stronger efficacy compared to bakuchiol and enhanced safety profile compared to retinol, SME may be the next generation of natural alternatives to retinoids.

## 1 Introduction

In cosmetic applications, vitamin A and its derivatives are among the most effective ingredients in products aiming to slow the aging process [1]. Their anti‐wrinkle properties promote keratinocyte proliferation, strengthen the protective function of the epidermis, reduce transepidermal water loss, protect collagen against degradation, and inhibit metalloproteinase activity. However, these positive effects come at a cost, with many users of retinol‐containing actives reporting irritation and redness of the skin. These negative effects can hinder acceptance by consumers and lead them to discontinue the use of otherwise highly effective products. Studies have shown that these effects are linked to retinol binding to retinoic acid receptors (RAR) in skin cells. This binding triggers gene transcription with a number of consequences, including suppression of sebum production [2, 3]. This mechanism of action may explain the dryness and irritation observed. Products that induce similar positive effects but bypass RAR may be safer and better tolerated in cosmetic applications.

Bakuchiol has emerged as a competitor to retinol, it has a different molecular structure [4]. It offers an improved tolerance profile, but with slightly weaker effects on some elements of comparison [5, 6, 7]. Bakuchiol is derived from *Psoralea corylifolia* and is related to the family of psoralenes or linear furanocoumarins—the molecules causing the irritant and photosensitizing effects of sap from fig, umbelifera such as parsley, and citrus fruits [8, 9]. Consequently, in addition to its reduced efficacy compared to retinol, bakuchiol may not be completely safe for daily application to the skin.

Milk thistle (*Silybum marianum* Gaertn.), sometimes also called wild artichoke, is a prickly plant native to Europe. It is used in traditional herbal remedies [10], mainly for its polyphenols (flavonolignans), which include silybin, isosylibin, silychristin, and silydianin [11, 12]. These antioxidant compounds are highly enriched in the seeds. Silybin is the most abundant biologically active compound extracted from milk thistle and the compound with the greatest effect [13]. It has been shown to impact several cellular pathways—with antioxidant properties as well as inflammation—and apoptosis‐modulating effects [14]—that protect cells from damage. Studies have shown that the pro‐apoptotic and anti‐inflammatory effects of silybin are linked to cytokine modulation, with effects on NF‐kB, Bcl‐2, and caspases [15]. These effects have been harnessed in the treatment of liver disease and to reduce side effects of treatments for gastrointestinal cancers and childhood leukemia [16, 17, 18]. These effects appear to be linked to increased susceptibility to apoptosis among cancer cells, but not healthy cells [19]. Silybin has also been shown to have an antimetastatic effect by targeting invasive behavior of cancer cells [10]. Mechanistic analyses revealed it to target signaling molecules that regulate the epithelial‐to‐mesenchymal transition, among other factors, in order to inhibit metastasis formation. These effects have been harnessed in topical applications, which have been shown to have a powerful effect in counteracting the dramatic skin damage caused by mustard gas [20] or exposure to ultraviolet light [21]. The latter effects are linked to reduced cell proliferation and enhanced expression of p53 and p21 proteins, protecting DNA against UV‐induced damage.

**FIGURE 1:**
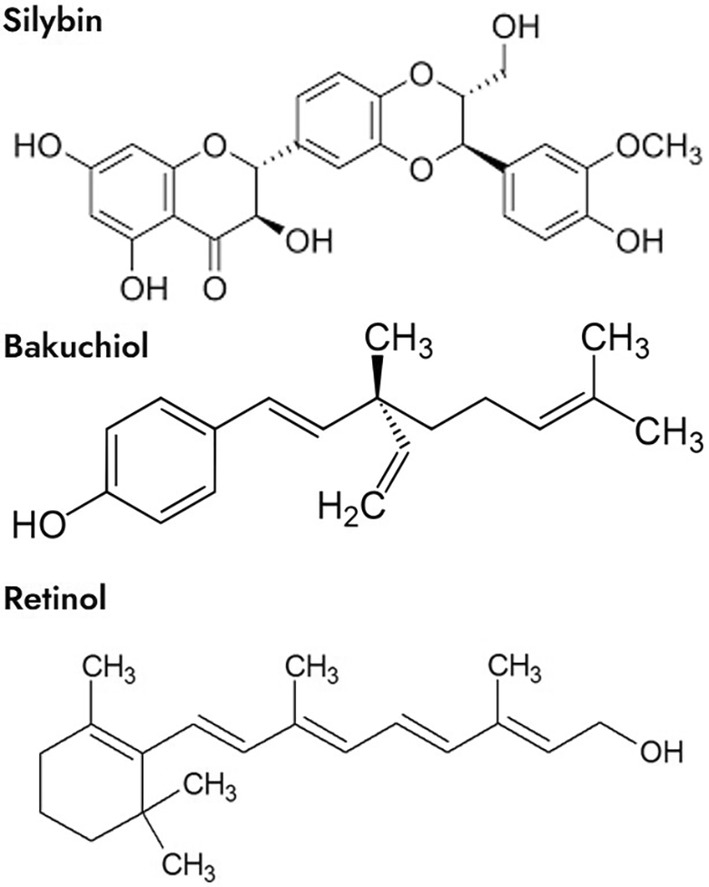
Chemical structures of silybin, bakuchiol, and retinol. The three compounds share a hydrocarbon backbone. Retinol and bakuchiol have a single aromatic or quasi‐aromatic element associated with a long chain. Silybin associates several aromatic modules and shares similar side chains with the other two molecules.

This activity profile makes silybin promising for use in cosmetic skin‐care applications. In this context, it is interesting to note that silybin shares some chemical side chains with retinol and bakuchiol (Figure 1). However, this chemical structure makes native silybin relatively insoluble in water and confers a low affinity for the skin. To circumvent this problem in oral formulations, the bioavailability was successfully enhanced by encapsulation in hybrid liposomes [22]. According to the results of this study, encapsulation enhanced stability and the downstream effects of treatment. Similarly, we developed a formulation with lecithin to minimize silybin's crystalline characteristics and facilitate its interaction with other biomolecules [23]. With our formulation, tests show it to provide better skin affinity, which should make the active elements more bioavailable to skin cells. We named this silybin‐liposomes SME and we aimed to evaluate its safe antiaging properties in comparison to retinol and bakuchiol, the natural retinoid challenger.

In this study, we used a combination of in vitro and ex vivo tests to analyze the potential effects of SME on skin cells and their receptors. We completed our study with a clinical trial. In all tests, we compared the effects of SME to those of the two main competitors, retinol and bakuchiol.

## 2 Materials and Methods

### 2.1 Test Solutions

Retinol 50C was 50% retinol solubilized in polysorbate 20 (INCI: retinol, polysorbate 20), a standard commercially available preparation. Retinol 50C was used at 0.2% meaning 0.1% pure retinol, the maximum dose allowed to limit side effects. Bakuchiol (INCI: bakuchiol) was a standard commercially available preparation and was tested at the same level as Retinol 50C. SME was prepared from *Silybum marianum* (INCI: lecithin and silybin) and was obtained by Indena Spa, Viale Ortles 12, Milan, Italy. It is obtained by complexing pure silybin, the most abundant and most active molecule found in milk thistle extract, belonging to the flavanolignan family, with phospholipids from lecithin in hydroethanolic solvents. The formation of complexes has been evidenced by IR and NMR spectra [23]. In the end, SME is composed of 29.7%–36.3% of silybin encapsulated into a phytosome (HPLC) and was used with a four times higher dose than both Retinol 50C and bakuchiol to reach a similar cost in formula. The phytosome formulation ameliorates the active molecules affinity for the skin and it has been shown to increase penetration and the kinetics if the delivery into the skin as well. Products were used at the concentrations indicated, and negative control samples corresponded to vehicle alone.

### 2.2 RARɣ Binding Tests

Human recombinant retinoic acid receptor gamma (RARɣ) receptor was purchased from ProteinOne. Coactivator recruitment assays were performed with a 25 nM purified receptor in Tris‐HCl buffer pH 7.4 for 16 h at 4°C. The three test compounds were used at the following concentrations: 0.3; 1; 3; 10; 30; 100; and 300 μg/mL. AM580 at 0.3 μM (Enzo, BML‐GR‐104) was used as a positive control for RARɣ activation. Complex formation was measured by spectrofluorimetry (excitation: 337 nm, emission: 620/665 nm) based on the TR‐FRET method. Evidence of RARɣ binding, with an increase in fluorescence by ≥ 50% relative to AM580 was considered possible RARɣ receptor agonist activity.

### 2.3 Measuring Antioxidant Activity

Primary cells were prepared from freshly isolated biopsies obtained from healthy donors undergoing abdominal surgery. Normal Human Epidermal Keratinocytes (NHEKs) were seeded in quadruplicate in 96‐well black glass‐bottomed plates precoated with type I collagen at 20 000 cells per well. Cells were incubated for 24 h in a complete medium (Epilife medium supplemented with HKGS) at 37°C under 5% CO_2_ in a humidified atmosphere. Test compounds were added to a complete medium at the following concentrations: Resveratrol 50 μM (positive control), SME 0.004 and 0.02 mg/mL (w/v), retinol and bakuchiol 0.001 and 0.005 mg/mL (w/v). Cells were incubated for 24 h under 5% CO_2_ in a humidified atmosphere before testing for activation. To test for reactive oxygen species, 2′,7′‐dichlorofluorescin diacetate (DCFH‐DA) probe (50 μM) was added to the wells for 30–40 min at 37°C. Cells were then washed twice with PBS, removing liquid carefully to avoid disturbing the cell layer. Oxidative stress was induced by applying tert‐butyl hydroperoxide solution (TBP) at 5 mM in PBS. The negative control was exposed to a PBS buffer. Fluorescence was measured at 525 nm in darkness, following excitation at 488 nm, using a microplate reader (TECAN). The study was performed in duplicate (*n* = 4 both times).

### 2.4 Ex Vivo Evaluation

#### 2.4.1 Culture and Treatment

Skin explants were obtained from a 58‐year‐old patient undergoing abdominal surgery. The study protocol was designed in line with ethical and regulatory requirements, the donor gave informed consent for the use of samples. Skin explants were prepared by cleaning the surface of the skin to remove oils, followed by a dehydrating bath in 70% ethanol before placing them in a Genoskin medium containing 1% antibiotic and 1% fungizone (Sigma). Multiple skin sections were prepared using a 6‐mm biopsy punch. Sections were placed on 3D‐bioprinted support, in contact with Genoskin medium (containing 1% antibiotic and 1% fungizone [Sigma]), for treatment. Intactness was verified by hematoxylin and eosin stain, after applying topical treatments with products for 5 days. Products were diluted in emulsion formula and used at the following concentrations: pure retinol at 0.1%, bakuchiol at 0.2%, and SME at 0.8%. A placebo treatment was included, corresponding to formula alone. Treatment and medium were renewed every day. Explants were incubated throughout the procedure at 37°C under a 5% CO_2_ humidified atmosphere. Treatments were performed twice in triplicate in preparation for the subsequent analyses.

#### 2.4.2 Sirius Red Staining

After treatment, skin explants were fixed in formalin (Sigma) for 48 h and then dehydrated overnight in a dehydrator (Leica). Dehydrated skin explants were embedded in paraffin and 4‐μm sections were prepared with a microtome (Leica). Paraffin was removed by treating sections with xylene. Samples were then dehydrated with graded ethanol solutions before staining with Sirius Red (Labo Modern) to reveal collagen networks. Stained samples were mounted on a thin glass slide and the structure of the dermis was observed in bright fields and under polarized light. In these conditions, collagen I appears red, and collagen III is in green. Relative proportions of each type of collagen were determined by quantification using ImageJ software.

#### 2.4.3 HABP Immunofluorescence

After treatment, skin biopsies were embedded in OCT (VWR) and stored at −80°C. Frozen skin sections were used to prepare 8‐μm sections, which were then stained with an antibody specifically detecting Hyaluronic acid binding protein (anti‐HABP, Merk). Antibody was diluted 1/100 in 1X TRIS buffer (VWR) with Bovine Serum Albumin (Sigma), and samples were exposed for 2 h at RT. Skin sections were then washed three times in 1X TRIS buffer before incubating with fluorescently labeled streptavidin (Alexa Fluor, Invitrogen) to reveal binding. DNA was stained with DAPI (Invitrogen). Stained sections were observed under fluorescence microscopy (Nanozoomer S60, Hamamatsu). Relative fluorescence was quantified for all images using ImageJ software and normalized relative to the DAPI signal.

### 2.5 Clinical Investigation

#### 2.5.1 INCI Formulations

All formulations were prepared with the same base. The placebo treatment consisted of the base alone, other treatments were added to the base according to standard cosmetic manufacturing procedures.

1. Placebo (base): Aqua/water, isodecyl neopentanoate, cetyl alcohol, glyceryl stearate, phenoxyethanol, peg‐75 stearate, ceteth‐20, steareth‐20, dimethicone, fragrance
2. SME 0.8%: Aqua/water, isodecyl neopentanoate, cetyl alcohol, glyceryl stearate, phenoxyethanol, lecithin, silybin, peg‐75 stearate, ceteth‐20, steareth‐20, dimethicone, fragrance
3. Bakuchiol 0.2%: Aqua/water, isodecyl neopentanoate, cetyl alcohol, glyceryl stearate, phenoxyethanol, peg‐75 stearate, ceteth‐20, steareth‐20, dimethicone, bakuchiol, fragrance
4. Retinol 50C 0.2% (corresponds to 0.1% pure retinol): Aqua/water, isodecyl neopentanoate, cetyl alcohol, glyceryl stearate, phenoxyethanol, peg‐75 stearate, ceteth‐20, steareth‐20, dimethicone, retinol50c, polysorbate 20, fragrance.

#### 2.5.2 Study Cohort and Protocol

Fifty‐seven volunteers (aged between 45 and 73, mean age: 59.4 ± 7.7 years) were recruited to the study. All volunteers met the inclusion and exclusion criteria defined in the protocol, including the presence of wrinkles on crow's feet and forehead areas. All subjects participating in the study gave their informed consent before starting the study. The study protocol was in compliance with the Declaration of Helsinki. During the study, volunteers applied a face cream formula twice daily for 56 days.

Concerning the repartition of groups for the study:

- Group 1 (SME): 14 volunteers,
- Group 2 (Retinol): 14 volunteers,
- Group 3 (Bakuchiol): 15 volunteers,
- Group 4 (Placebo): 14 volunteers.

Four formulations were used, a base common to all the preparations (placebo), or one of three active formulations, with SME at 0.8%, bakuchiol at 0.2%, or retinol 50C at 0.2% (meaning pure retinol at 0.1%) added to the base.

#### 2.5.3 Visia CR2.3 Analysis to Measure Wrinkle Thickness and Skin Redness

Digital photographs of the volunteers' faces and periorbital areas were taken on D0 and after the study period of 56 days using a Visia CR 2.3 (Canfield imaging systems). Posttreatment images were compared to D0 images directly on the data‐processing screen using an overlay visualization of the images at each acquisition time. The Visia CR 2.3 can take pictures under different types of illumination and allows very rapid image capture. A series of photos taken under multispectral imaging and analysis provide visual information on the appearance of the skin. Photos were taken of the forehead and each side of the face. Wrinkles in periorbital areas, and skin redness on the face were measured after 56 days.

#### 2.5.4 AEVA‐HE Analysis to Measure Wrinkle Numbers and Circumference on Forehead

The depth, length, and number of wrinkles on the forehead were measured using a contactless AEVA‐HE system (Eotech) with 250 sensors. This system is based on a fringe projection unit and uses light associated with stereometry to provide high‐resolution 3D scanning. Volunteers were installed using VisioTOP‐500 benches to ensure accurate and stable positioning and re‐positioning between the different measuring times. Skin wrinkle data were measured on D0 and D56, and the results were compared.

#### 2.5.5 Collagen Measurement Using SIASCOPE

To measure collagen levels in volunteers' skin, a portable scanning device (SIAscope) connected to Siametrics software was used. The SIAscope was placed in contact with the skin, and the skin was illuminated. Light that is not reflected or scattered from the surface is transmitted into the top layers of the skin, where it can be absorbed by the melanin in the epidermis or transmitted to the dermis, where it will be absorbed by hemoglobin in the blood vessels. Scattering also occurs in the dermis when the light interacts with collagen, resulting in some of the light re‐emerging at the surface. Interpretation of the combination of wavelengths reflecting back to the SIAscope is used to produce SIAscans; based on inbuilt proprietary mathematical models of skin optics. Collagen and its distribution were analyzed on the oval part of the face, above the chin, after 56 days of application. Results were normalized relative to measurements taken on D0.

#### 2.5.6 Skin Barrier and Hydration Using Corneometer

Changes to hydration of the stratum corneum were measured based on its electrical characteristics. For this study, three measurements were performed on the forehead (middle) at D56 with a Corneometer CM 825 (Courage & Khazaka electronics). The probe linked to a condenser was used to apply a constant pressure to the tegument to ensure reliable and reproducible measures. The measurement time was recorded. The Corneometer CM 825 was controlled by “CLS” software. Results at D56 were normalized relative to measurements performed at D0.

### 2.6 Statistical Analysis

#### 2.6.1 In Vitro/Ex Vivo Experiments

For all studies, a Shapiro–Wilk test was used to verify whether raw data were normally distributed. Mean values of normally distributed data were compared using an unpaired Student's *t*‐test. For nonnormally distributed data, a Mann–Whitney *U* test was used.

#### 2.6.2 In Vivo Experiments

All raw data from in vivo experiments were analyzed by Shapiro–Wilk test in order to determine whether they were normally distributed. Normally distributed data were analyzed using parametric paired or unpaired Student's *t*‐tests. Nonnormally distributed data were analyzed using a nonparametric Wilcoxon test or nonparametric Mann–Whitney test.

#### 2.6.3 Statistical Significance

Whatever the statistical test, or type of experiment, significant results were classed as follows: #*p* < 0.1, *p < 0.05, **p < 0.01, ***p < 0.001 and ****p < 0.0001. Self‐assessments were analyzed by the contingency method, applying a chi‐squared test, with **p < 0.01. All the figures are presented with mean ± SEM.

## 3 Results

### 3.1 RAR Gamma Activation

**FIGURE 2:**
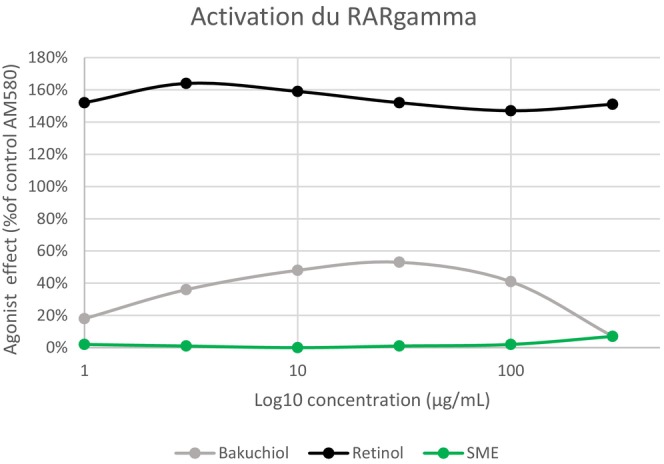
Binding to the RARɣ receptor was assessed using a recombinant receptor produced in *Escherichia coli*. Agonistic effects were measured by an FRET method, with spectrofluorimetry readout (excitation: 337 nm, emission: 620/665 nm). An increase in fluorescence by ≥ 50% relative to AM580 was considered possible RARɣ receptor agonist activity.

RARɣ is the main retinoid receptor in the skin [24], and its activation has been linked to many of the side effects of retinol‐containing products. To determine whether SME would have similar side effects, we measured the agonist effect of the three test solutions—retinol, bakuchiol, and SME—using recombinant human RARɣ in functional assays. RAR gamma‐ligand‐binding domain (RARɣ‐LBD) was produced in *Escherichia coli* and labeled on the ligand‐biding domain. Purified receptors were incubated with each active at a range of concentrations and AM580, a selective RARɣ agonist, as a positive control. The activation measured with each of the test solutions was normalized relative to AM580 activation (Figure 2).

As expected, given its known mode of action, retinol strongly activated RARɣ. Its agonist effect was even higher than that measured with AM580, resulting in activation levels exceeding 140% between 1 and 300 μg/mL. Bakuchiol also induced some RARɣ activation (~ 55%). However, exposure to between 1 and 300 μg/mL SME resulted in almost no activation of the receptor (Figure 2).

### 3.2 In Vitro Reactive Oxygen Species Production

**FIGURE 3:**
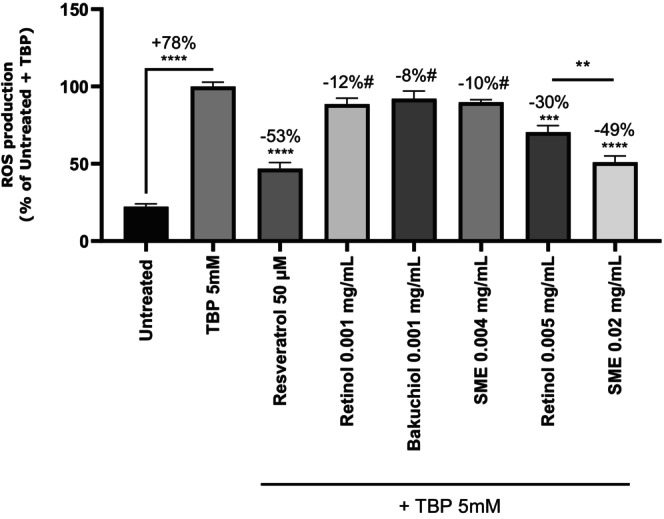
SME protects NHEK cells against intracellular ROS production. The probe 2′,7′‐dichlorofluorescin diacetate (DCFH‐DA) (50 μM) was added to NHEK cultures before inducing oxidative stress with tert‐butyl hydroperoxide solution (TBP); 5 mM in PBS. The negative control was exposed to PBS buffer. Fluorescence was measured at 525 nm in darkness, following excitation at 488 nm, on a microplate reader. #*p* < 0.1, **p < 0.02, ***p < 0.001, ****p < 0.0001.

Skin aging is known to be linked to the production of reactive oxygen species (ROS) which can be counteracted by antioxidants. Orally administered silybin is reported to have an antioxidant effect [25]. To test whether this was also the case with topical applications, we treated primary Normal Human Epidermal Keratinocyte (NHEK) cultures derived from human skin explants with SME at 0.004 and 0.02 mg/mL, or retinol or bakuchiol at 0.001 and 0.005 mg/mL. After 24 h, oxidative stress induced by TBP was measured by fluorescence (Figure 3). Induction of oxidative stress with TBP 5 μM increased ROS production by 78%. Resveratrol, the positive control, reduced ROS production by 53%, thus validating the experimental setup.

Treatment with bakuchiol reduced ROS production (8% reduction with 0.001 mg/mL) but induced significant cytotoxicity from 0.005 mg/mL. Consequently, this dose with this substance were excluded from further analysis. The other two test substances induced no toxicity. Treatment with retinol and SME led to dose‐dependent reductions in ROS levels, with significant differences between the two treatments (Figure 3).

### 3.3 Alterations to the Collagen Profile Following Ex Vivo and In Vivo Treatments

**FIGURE 4:**
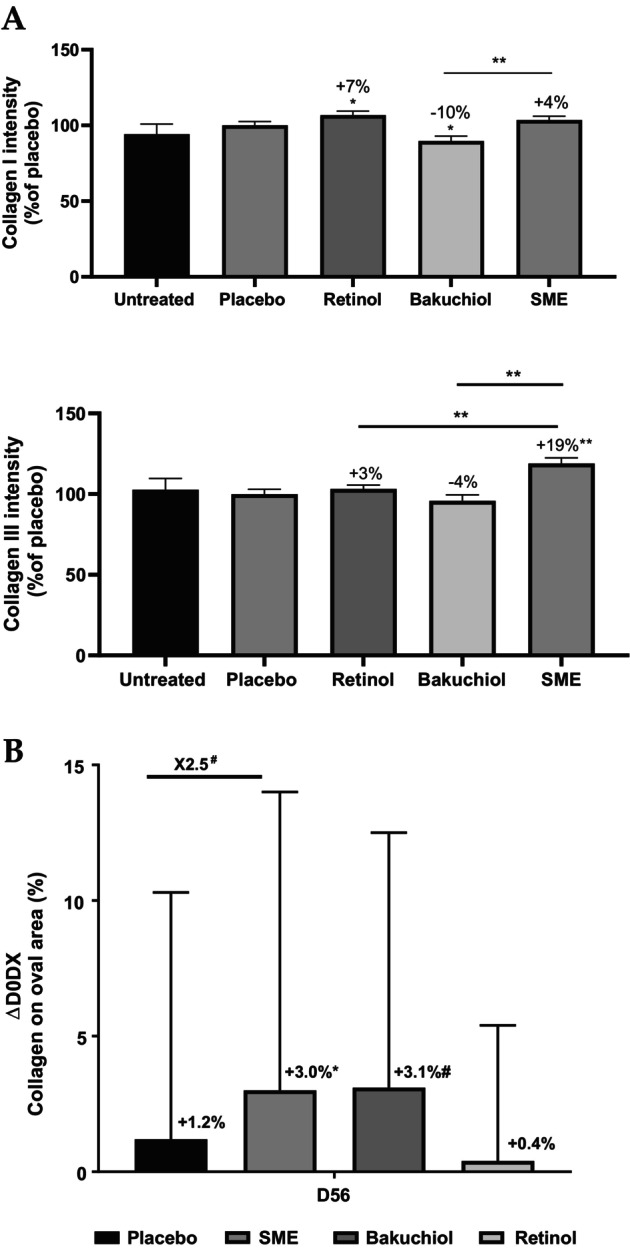
SME increases collagen production ex vivo and in vivo. (A) Upper panel: relative proportions of collagen I were determined by quantifying the red channel. Lower panel: relative proportions of collagen III were determined by quantifying the green channel. Channels were quantified using ImageJ software. Data were normalized relative to the placebo treatment (100%). (B) Collagen content of volunteers' skin after applying products twice daily for 56 days. Collagen content was measured on the oval part of the face by siascopy. #*p* < 0.1, *p < 0.05, **p < 0.01.

The skin's elasticity is closely linked to the collagen network linking the cells together. With advancing age, the nature of this network changes, and the fibers become less elastic. The antiaging properties of the three test compounds were first assessed based on collagens I and III quantifications using an ex vivo model. Human skin explants were treated with pure retinol at 0.1%, bakuchiol at 0.2%, SME at 0.8%, or the placebo formulation for 5 days. Skin sections were stained with Sirius Red and analyzed under polarized light to reveal collagen I (red) and collagen III (green) fibers. The amounts of the two types of collagen were determined relative to the placebo treatment. Both retinol and SME increased the presence of collagen I in explants. In contrast, bakuchiol led to a significant reduction in collagen I levels by 10%. SME significantly increased its content by 14% in comparison to bakuchiol treatment (Figure 4A).

To determine whether similar effects were observed in vivo, we performed siascopy on volunteers in our clinical trial, after application of the products twice daily for 56 days. This intracutaneous spectrophotometric method provides information based on how light interacts with the skin, thus making it possible to determine the distribution of chromophores in the skin: melanin, hemoglobin, and collagen. Chromophores can be detected at up to 2 mm under the skin. In the dermis, light is refracted following interaction with collagen. The results show that retinol had no effect, but both bakuchiol and SME had a stimulating effect on collagen production (Figure 4B). The effect was significant only for SME.

### 3.4 Hyaluronic Acid Production Measured by HABP Immunostaining in an Ex Vivo Model and Increased Hydration In Vivo

Hyaluronic acid (HA) is one of the skin's natural hydrating agents. The skin loses moisture content as it ages. Consequently, any treatment that can influence HA production should have a hydrating and plumping effect. Human skin explants were treated for 5 days with the three active compounds in the same conditions as for collagen measurements. Frozen skin sections were then stained with an antibody specific for hyaluronic acid binding protein (HABP) to reveal the presence of HA, and DNA was counterstained with DAPI. HABP staining was quantified and normalized relative to DAPI staining.

**FIGURE 5:**
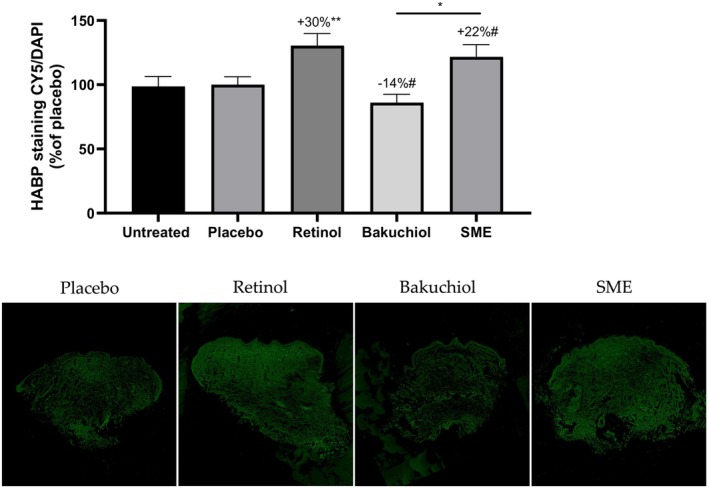
SME increases hyaluronic acid production in skin explants. Skin biopsies were embedded in OCT and frozen at −80°C before preparing 8 μm sections. Sections were immunostained to detect hyaluronic acid binding protein and counterstained with DAPI. Stained sections were observed under fluorescence microscopy. Relative fluorescence was quantified for all images using ImageJ software, and normalized relative to the DAPI signal (up) and illustrative fluorescence pictures were added (down). #*p* < 0.1, *p < 0.05, **p < 0.01.

After treatment with retinol and SME for 5 days, significant increases in HABP were measured compared to the placebo, at 30% and 22%, respectively. In contrast, treatment with bakuchiol led to a 14% reduction in HABP immunofluorescence compared to the placebo. The difference in effect between SME and bakuchiol was also significant (Figure 5).

**FIGURE 6:**
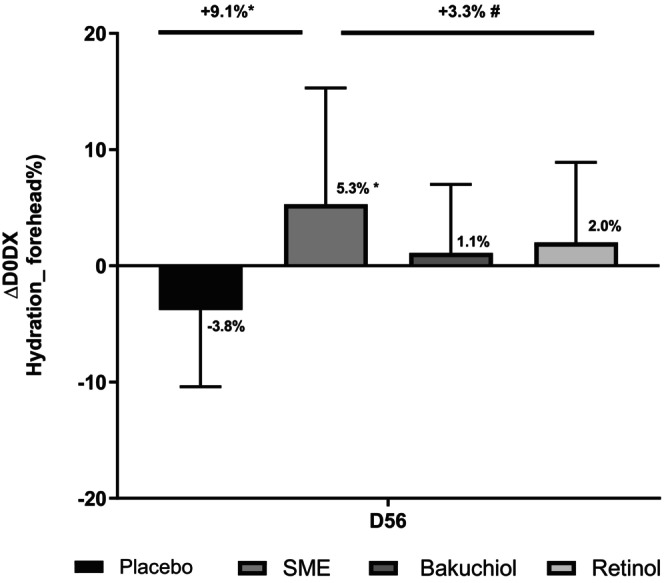
SME significantly improves skin hydration in vivo. Volunteers applied topical skin cream twice daily for 56 days. Skin hydration was analyzed on the face using a Corneometer. #*p* < 0.1, *p < 0.05.

In our clinical study, we assessed hydration after applying products twice daily for 56 days. We analyzed this parameter based on the electrical characteristics of volunteers' skin, using corneometry measurements (Figure 6). The three actives provided improved hydration relative to D0 but the effect of SME (+5.3%) was considerably greater than that of bakuchiol (+1.1%) or retinol (+2.0%). SME was the only treatment to induce a significant effect (*p* < 0.05). The placebo had a negative, although not statistically significant, effect on hydration relative to D0 (−3.8%). The effect of SME at D56 was also significant relative to the placebo (*p* < 0.05).

### 3.5 Clinical Investigation

Our clinical study involved 57 female volunteers aged 45–73 years. The objective was to analyze the antiaging properties of 0.8% SME, and compare them to the two benchmark actives: 0.1% pure retinol and 0.2% Bakuchiol. The active compounds were included in otherwise identical formulations and applied to the face twice daily for 56 days. A placebo formulation was also included. Wrinkles on the face, in the periorbital region, and on the forehead were studied. Skin tolerance and the skin barrier—based on water content—were also analyzed. Skin redness was assessed as well in similar application conditions.

#### 3.5.1 SME Reduces Wrinkle Thickness in the Periorbital Area and Over the Whole Face

**FIGURE 7:**
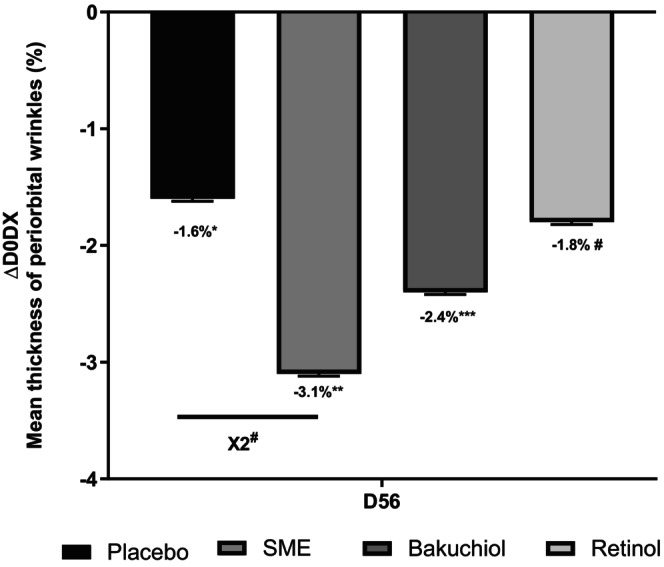
Volunteers applied topical skin cream twice daily for 56 days. Wrinkle thickness was measured on the periorbital area at D0 and D56 using the Visia CR2.3. #*p* < 0.1, *p < 0.05, **p < 0.01, and ***p < 0.001.

Wrinkles in the periorbital area were measured using an imaging system after 56 days' application of treatments twice per day. All the formulations, including the placebo, produced some reduction in wrinkle thickness relative to D0. Compared to the placebo treatment, only 0.8% SME produced a significant effect—reducing wrinkle thickness almost twofold (Figure 7).

**FIGURE 8:**
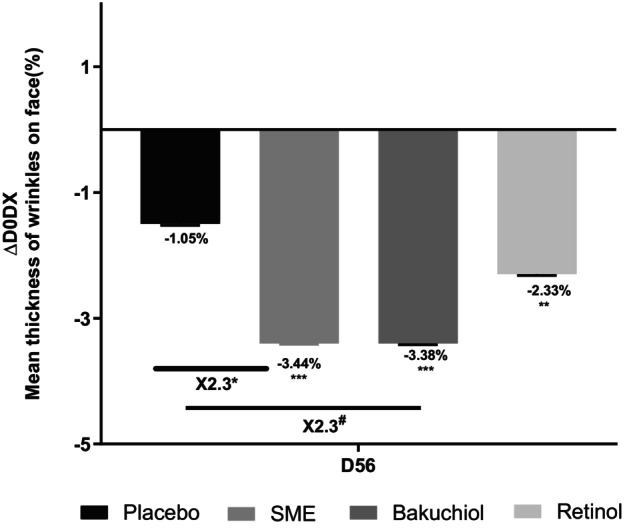
Volunteers applied topical skin cream twice daily for 56 days. Wrinkle thickness was measured on the whole face at D0 and D56 using the Visia CR2.3. #*p* < 0.1, *p < 0.05, **p < 0.01, and ***p < 0.001.

Imaging analysis of wrinkles on the whole face produced similar results, although in this case, 0.8% SME and 0.2% bakuchiol had similar effects—reducing wrinkle thickness 2.3‐fold with respect to the placebo treatment (*p* < 0.05 and *p* < 0.1, respectively). The effect with 0.1% pure retinol was smaller and not significantly different from the placebo effect (Figure 8).

#### 3.5.2 SME and Retinol Reduce Wrinkle Number and Circumference on the Forehead

**FIGURE 9:**
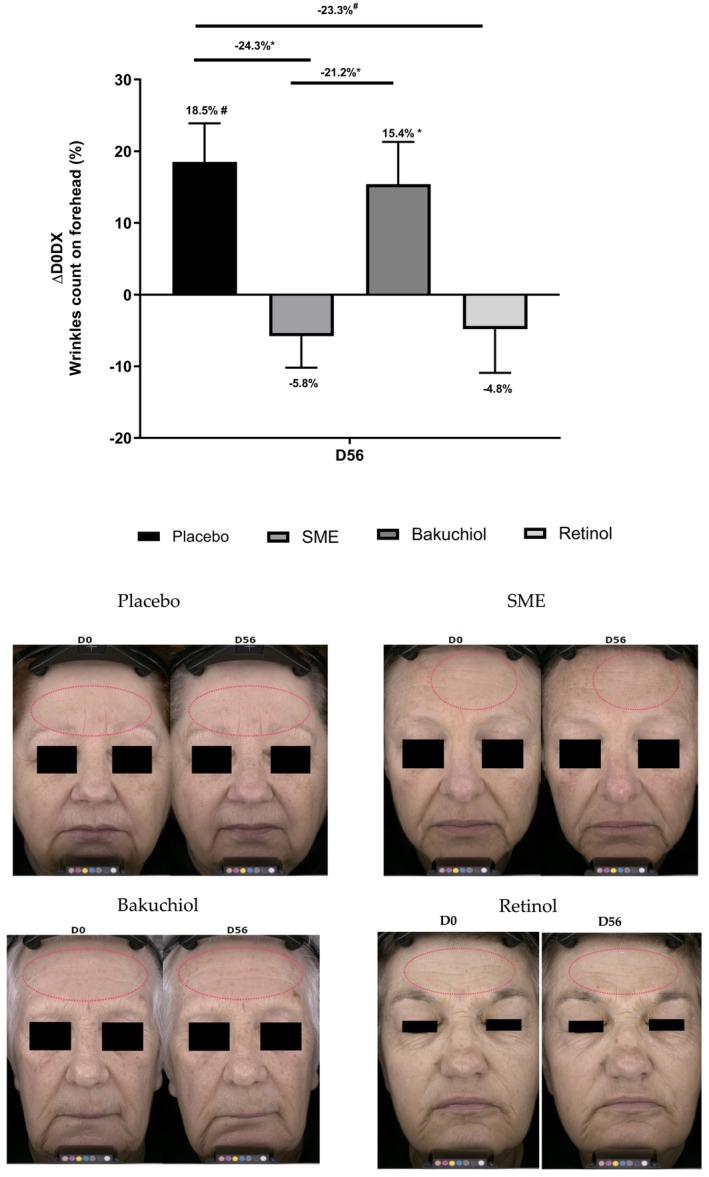
Volunteers applied topical skin cream twice daily for 56 days. Wrinkle count was measured on the forehead using the AEVA‐HE. Upper panel: evolution in wrinkle count compared to D0. Lower panel: photographs were taken on D0 and D56, here to measure wrinkle count on the forehead.

The forehead is a key area for wrinkle formation, we, therefore, counted the number of wrinkles on the forehead at the end of the treatment regime (56 days, twice‐daily applications). In this analysis, bakuchiol and the placebo had similar effects—with a slight increase in wrinkle number on D56. The other two treatments produced a marked, although not significant, improvement relative to D0: 5.8% reduction with 0.8% SME and 4.8% reduction with 0.1% pure retinol. Compared to the placebo treatment, the reduction in numbers of wrinkles was significant in both cases (*p* < 0.05 for SME, *p* < 0.1 for retinol) (Figure 9).

#### 3.5.3 SME Provides Better Skin Tolerance Than Retinol or Bakuchiol

**FIGURE 10:**
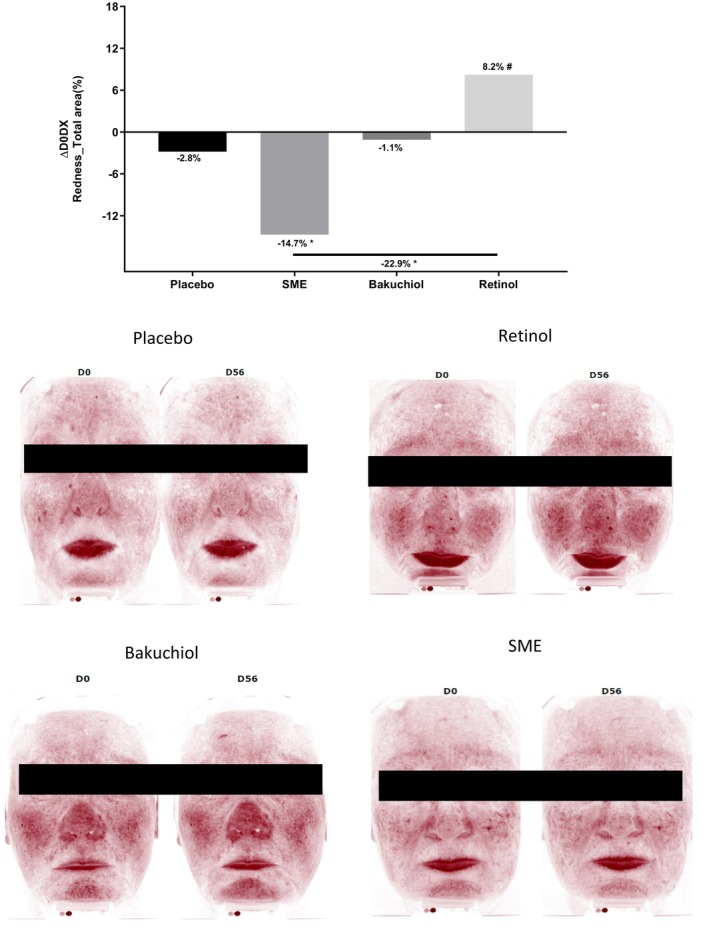
SME induces significantly less skin redness. Volunteers applied topical skin cream twice daily for 56 days. Skin redness was measured on the whole face using the Visia CR2.3. Upper panel: redness quantified for the different skin treatments. Lower panel: photographs were taken on D0 and D56, only the red channel was treated to get an overall indication of redness. #*p* < 0.1, *p < 0.05.

Increased skin redness is one of the main side effects of retinol use. To compare the effect of the three treatments, we measured this parameter using a specifically adapted imaging technique (Figure 10). A comparison of skin redness between D0 and D56 revealed increased redness following the application of retinol (+8.2%, *p* < 0.1), as expected. The placebo and bakuchiol induced neither an increase nor a decrease in redness. In contrast, in volunteers applying SME, redness was markedly and significantly reduced relative to D0 (−14.7%, *p* < 0.05).

#### 3.5.4 Subjective Assessments

As a large part of how cosmetic products work is based on the users' perception, we also included a subjective assessment of efficacy.

**FIGURE 11:**
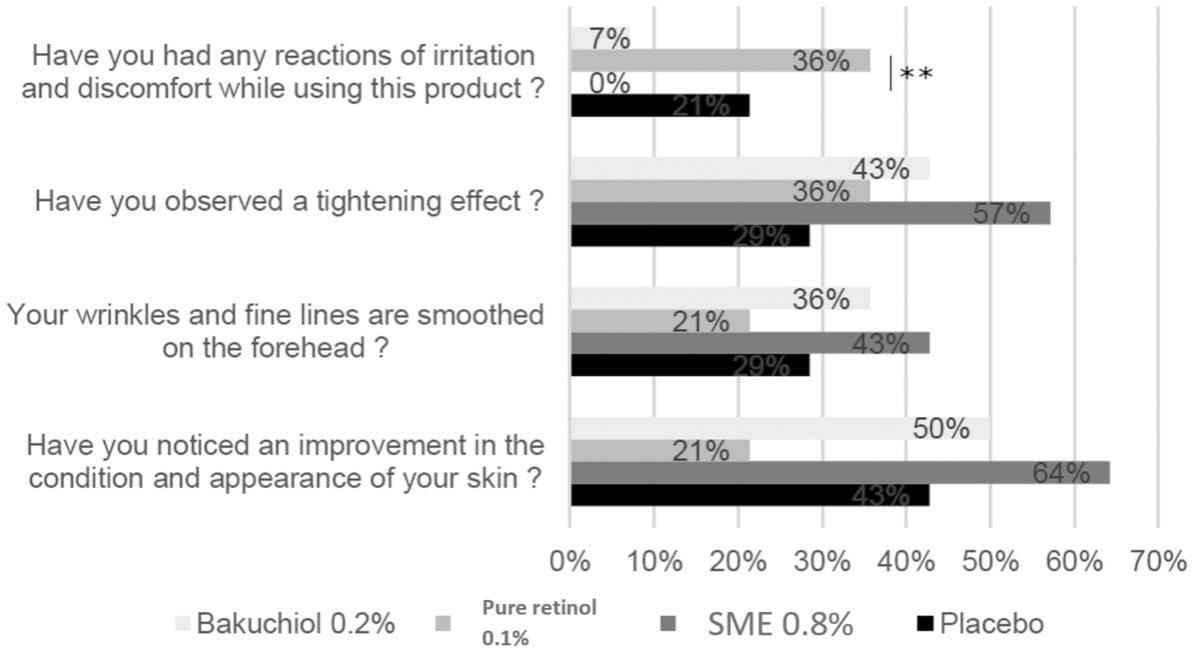
Subjective assessment of efficacy. Volunteers applied topical skin cream twice daily for 56 days. They then answered a questionnaire to determine the observed effects. **p < 0.01.

From the responses to this questionnaire, in terms of the positive effects—tightening effect, smoothing of fine lines, and improved skin condition—the differences between the treatments were not statistically significant. In contrast, for the question related to irritation and discomfort, the SME formulation provided the best performance, with 0% of participants reporting a reaction (Figure 11). This result was significantly better than the reports from participants applying the retinol formulation—where 36% reported irritation and/or discomfort (*p* < 0.01).

## 4 Discussion

The aim of this study was to measure the effects of a new cosmetic ingredient with a similar biological activity but distinct and safer mode of action to retinol. Performance was compared to a retinol formulation and to the current best competitor, bakuchiol, in in vitro, ex vivo, and clinical trials.

The positive antiwrinkle effects of retinol‐containing cosmetic products are associated with side effects including skin redness and irritation. These negative effects can reduce adherence to continued use of products. Studies have linked them to antagonistic interactions between retinol and a number of cellular receptors. For example, retinoids activate the irritant TRPV1 receptor [26]. This receptor also binds capsaicin—the compound giving chili peppers their heat—which explains why some users report sensations of heat or irritation when using retinoid‐containing formulations. Interactions between retinoids and RAR have also been demonstrated [27]. The main competitor to retinol, bakuchiol, is better tolerated than retinol, but is slightly less effective [5]. The comparative in vitro tests performed here indicated that both retinol and bakuchiol bound to the RAR receptor. Previous reports indicate that this binding has an antagonistic effect (Chaudhuri and Bojanowski, [6]). The lower binding observed with SME suggests that it would induce fewer side effects than either of the two current actives.

Retinol and retinol‐like compounds are particularly used to correct cutaneous photodamage. One effect of UV exposure is to induce the production of ROS—a by‐product of mitochondrial metabolism. ROS can induce cell damage throughout the body, but skin cells are particularly prone given their direct and continuous exposure to oxygen in the air. In addition, exposure to environmental pollutants has an exacerbating effect on ROS production [28]. Any product that protects skin cells from the deleterious effects of ROS should have a positive antiaging impact. Retinol has been shown to reduce ROS production [29], but the effect observed here in skin sections, with SME was greater, with 0.02 mg/mL having an effect equivalent to the positive control Resveratrol 50 μM.

Structurally, the cells in the skin are held together by an extracellular matrix, composed largely of collagen [30]. Several types of collagen exist, and the collagen profile of the skin changes with age, leading to a looser network and reduced elasticity. Cosmetic products that influence this network will have a longer‐lasting effect on the skin. Like retinol, bakuchiol is reported to target collagen and extracellular matrix synthesis enzymes [6]. However, in our ex vivo tests, we observed a reduction in collagen I and collagen III production compared to the control following the application of bakuchiol. In contrast, both retinol and SME increased collagen III production. The ratio of collagen III to collagen I is an indication of the “youth” of the network [31]. Retinol stimulated production of both types of collagen to similar extents, whereas both bakuchiol and SME had a stronger effect on collagen III production than on collagen I, resulting in higher ratios. This increased production could alter the collagen I:collagen III ratio, toward a younger skin profile, and should lead to improved skin elasticity. It has been recently described that *Silybum marianum* inhibited collagenase activity, which confirms our findings [32].

Reduced ROS production combined with restored elasticity provides protection against cell death and sagging, but another well‐known effect of age on the skin is increased dryness. HA has emerged as a veritable hero molecule in this context. It can be applied topically to capture and retain moisture in the upper dermal layers, but it is also produced directly by the skin cells. Products that can influence this production have a longer‐lived hydration effect than any topical application. Retinol has a stimulating effect on HA production [33], which was confirmed here in ex vivo studies. SME had an almost equivalent stimulating effect. *Silybum marianum* has been highlighted to inhibit hyaluronidase activity, which supports our findings [32].

This potential hydrating effect was confirmed in vivo with Corneometer measurements. Thus, the electrical characteristics of the stratum corneum—which behaves like a dielectric body—indicated elevated skin hydration following the application of SME. The effect was greater than that observed with either of the other two products tested.

All these results are very promising, but the real test of how any cosmetic product will perform in the market comes from user feedback. In our clinical trial, users reported significantly higher tolerance to an SME‐containing formulation than to the other formulations, including the placebo. We were surprised to observe higher levels of reported discomfort with the placebo treatment, with 21% of subjects reporting some issues. This could be a “placebo effect,” as volunteers would be aware of the side effects associated with retinol, but were blinded as to the composition of the product they applied. This type of effect is notoriously difficult to demonstrate. However, the comparative results for volunteers using the formulations containing bakuchiol or SME clearly show that tolerance is improved when using these products, with only 7% of users reporting discomfort with bakuchiol, and none reporting discomfort following application of the SME formulation. This improved subjective tolerance was corroborated by objective, automated, measurements of skin redness.

This study has certain limitations. In the RAR binding assay, the divergent results made it impossible to determine the EC50 value for each active. It was only possible to determine the EC50 for bakuchiol (15.6 μg/mL). In the clinical trial, the sample size was relatively small, and the study was done at a single centre. However, the number of study participants was comparable to that of similar studies pitting bakuchiol against retinol [5] and greater than that used in early trials of topical retinoids [34, 35].

## 5 Conclusions

The aim of this study was to measure the effects of a new cosmetic ingredient with a similar biological activity but a distinct and safer mode of action compared to retinol. Taken together, our in vitro, ex vivo, and in vivo results suggest that SME is a promising ingredient for inclusion in products aiming to improve skin condition in an aging population. It is better tolerated than retinol and more effective than bakuchiol.

## Author Contributions

Conceptualization: Cloe Boira, Emilie Chapuis, Amandine Scandolera, and Romain Reynaud. Methodology: Cloe Boira, Emilie Chapuis, Jean Tiguemounine, Amandine Scandolera, and Romain Reynaud. Formal analysis: Cloe Boira, Emilie Chapuis, Laura Lapierre, Amandine Scandolera, and Romain Reynaud. Investigation: Cloe Boira, Emilie Chapuis, Laura Lapierre, Amandine Scandolera, and Romain Reynaud. Writing – original draft preparation: Cloe Boira. Writing – review and editing: Cloe Boira, Emilie Chapuis, Laura Lapierre, Amandine Scandolera, and Romain Reynaud. All authors have read and agreed to the published version of the manuscript.

## Ethics Statement

The study was conducted in accordance with the Declaration of Helsinki. We do not have any specific ethical agreement for this case as, according to French regulations, it does not meet the criteria requiring evaluation by an ethics committee.

Indeed, according to French regulations, the evaluation of a cosmetic ingredient does not require approval from an ethics committee. However, the clinical study is conducted in accordance with good laboratory practices and the Helsinki agreements and provides the following information:

1. A toxicology certificate regarding the applied formulas.
2. A protocol describing the research objective, inclusion and exclusion criteria, measurement methods used, volunteer recruitment methods, methods of anonymizing volunteers, and methods of collecting and securing personal data.
3. Informed consent is provided to each volunteer. It describes the study objective and the means employed in an understandable manner as well as compensation and emergency contact numbers.

Only protocols involving “interventional” analysis methods are subject to ethical approval in France. If this is the case, the dossier is submitted to the “Comité de Protection des Personnes” (Ethics Committee) as well as internal IRB. The study mentioned in this article does not require this because no “interventional” measures are performed.

## Consent

Informed consent was obtained from all subjects involved in the study.

## Conflicts of Interest

The authors declare no conflicts of interest.

## Data Availability

The data that support the findings of this study are available from the corresponding author upon reasonable request.
